# Monocyte-neutrophil entanglement in glioblastoma

**DOI:** 10.1172/JCI163451

**Published:** 2023-01-03

**Authors:** Dinorah Friedmann-Morvinski, Dolores Hambardzumyan

**Affiliations:** 1 School of Neurobiology, Biochemistry and Biophysics, George S. Wise Faculty of Life Sciences, and; 2 Sagol School of Neuroscience, Tel Aviv University, Tel Aviv, Israel.; 3 Department of Oncological Sciences, Tisch Cancer Institute, and; 4 Department of Neurosurgery, Icahn School of Medicine at Mount Sinai, New York, New York, USA.

## Abstract

Glioblastoma (GBM) is the most belligerent and frequent brain tumor in adults. Research over the past two decades has provided increased knowledge of the genomic and molecular landscape of GBM and highlighted the presence of a high degree of inter- and intratumor heterogeneity within the neoplastic compartment. It is now appreciated that GBMs are composed of multiple distinct and impressionable neoplastic and non-neoplastic cell types that form the unique brain tumor microenvironment (TME). Non-neoplastic cells in the TME form reciprocal interactions with neoplastic cells to promote tumor growth and invasion, and together they influence the tumor response to standard-of-care therapies as well as emerging immunotherapies. One of the most prevalent non-neoplastic cell types in the GBM TME are myeloid cells, the most abundant of which are of hematopoietic origin, including monocytes/monocyte-derived macrophages. Less abundant, although still a notable presence, are neutrophils of hematopoietic origin and intrinsic brain-resident microglia. In this Review we focus on neutrophils and monocytes that infiltrate tumors from the blood circulation, their heterogeneity, and their interactions with neoplastic cells and other non-neoplastic cells in the TME. We conclude with an overview of challenges in targeting these cells and discuss avenues for therapeutic exploitation to improve the dismal outcomes of patients with GBM.

## Introduction

Considering the variety of unique and complex brain tumor microenvironments, this Review will mainly focus on research done in glioblastoma (GBM); results from extracranial tumors will occasionally be used for comparison, for outlining differences, or for extrapolation of understudied areas, such as neutrophils, for thought-provoking discussions. Since many excellent recent reviews have discussed the reciprocal interactions between tumor cells, monocyte-derived macrophages (MDMs), and microglia in great detail, we will not focus on this aspect here (1–5); instead, we will concentrate on neutrophil interactions with tumor cells, and myeloid cell interactions with other non-neoplastic cells in the tumor microenvironment (TME).

## Inter- and intratumor heterogeneity of GBM

**Figure 1:**
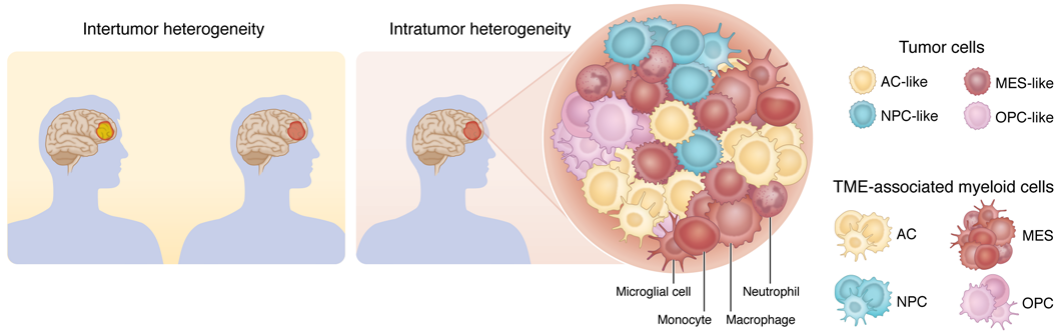
Inter- and intratumor heterogeneity of neoplastic and non-neoplastic compartments of GBM. Genetic and molecular heterogeneity in GBM occurs at multiple levels: between patients (intertumor) and within the tumor from the same patient (intratumor). Inter- and intratumor heterogeneity exists both in neoplastic cells and in the tumor microenvironment, especially in the myeloid compartment.

In the last two decades, numerous studies using high-dimensional technologies have revealed the high degree of inter- and intratumor heterogeneity of GBM. Initially, The Cancer Genome Atlas (TCGA) initiative, based on bulk RNA sequencing (RNA-Seq), provided robust gene expression–based identification of three GBM subtypes: proneural (PN), mesenchymal (MES), and classical (CL) (6–8). These subtypes are not mutually exclusive and are determined by the dominant transcriptional patterns at the time and place of tumor resection. With respect to the TME, it was shown that the MES signature and neurofibromin 1 (NF1) deficiency resulted in enrichment of myeloid cells, specifically microglia/macrophages (9, 10). Subsequent studies with more advanced single-cell RNA-Seq (scRNA-Seq) showed multiple transcriptional subtypes coexisting within a single tumor whose relative proportions can evolve over time, or in response to therapy (11, 12). An example of this is that when tumors with the PN signature are treated with radiation therapy, they can transition to the MES signature, a process referred to as PN to MES shift; and STAT3 has been shown to be an essential player in this process (13–15). Recently, using scRNA-Seq, malignant cells in human GBM were catalogued into four potentially plastic cellular states: neural progenitor cell–like (NPC-like), oligodendrocyte progenitor cell–like (OPC-like), astrocyte-like (AC-like), and mesenchymal-like (MES-like) (16). Subsequent studies showed that, in fact, macrophages induce MES cellular states in tumor cells, which is associated with corresponding increases in the MES program of macrophages themselves and increased cytotoxicity of T cells (17, 18). These studies clearly illustrate a reciprocal interaction between tumor cells and macrophages that influences expression profiles of both populations, and demonstrate that intratumor heterogeneity in neoplastic cells is determined not only by intrinsic mechanisms but also by extrinsic influences coming from TME cells. Therefore, inter- and intratumor heterogeneity in the neoplastic compartment is also present in the TME (Figure 1). Considering the plasticity of tumor cells with various gene signatures, it is essential to understand their role in influencing myeloid cell recruitment and shaping myeloid cell function in order to design effective therapies that work across multiple subtypes or cellular states of GBM.

## Ontogeny and destiny of neutrophils and monocytes

**Figure 2:**
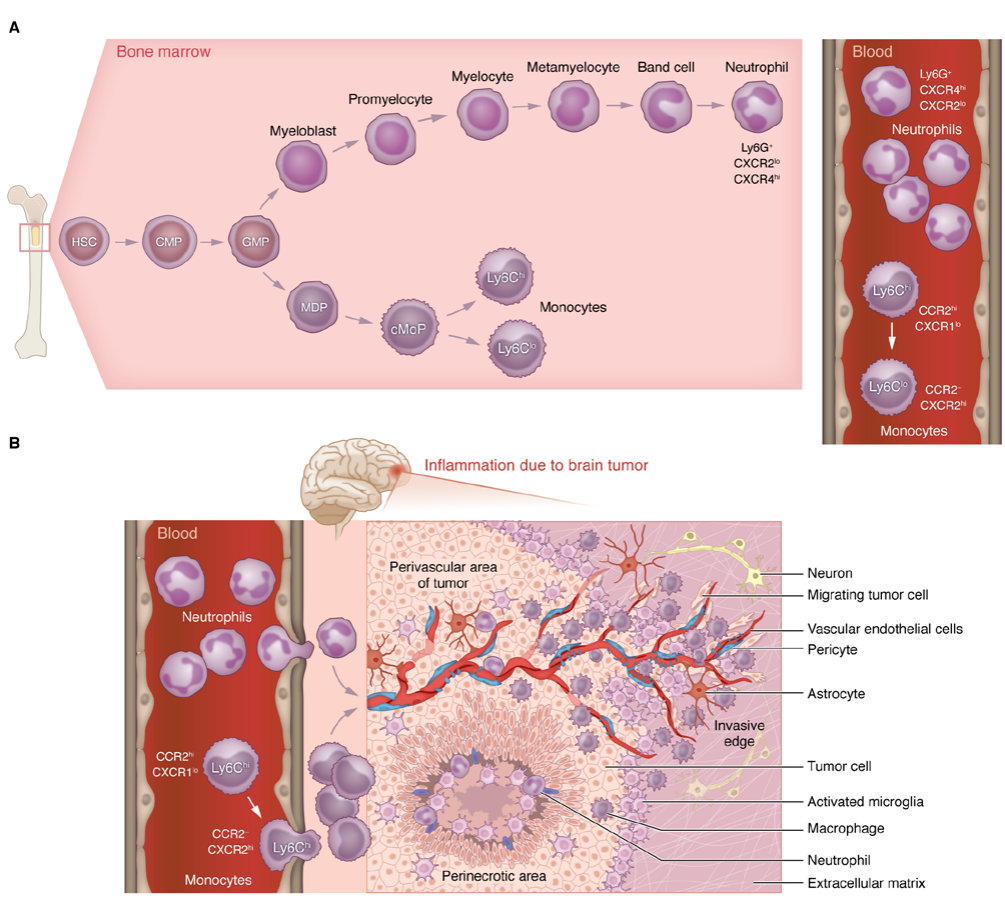
Monocyte and neutrophil origins in health and GBM. (**A**) Granulopoiesis and monocytopoiesis in mice. Monocytes and neutrophils are continuously generated in the bone marrow (BM) from hematopoietic stem cells (HSCs) via common myeloid progenitors (CMPs), giving rise to granulocyte-monocyte progenitors (GMPs). GMPs, via macrophage and dendritic cell (DC) precursor (MDP) and common monocyte progenitor (cMoP) cells, then give rise to functionally distinct Ly6C^hi^CCR2^hi^CXCR1^lo^ and Ly6C^lo^CCR2^–^CXCR1^hi^ monocyte subsets that enter the blood circulation. GMPs, under the control of the granulocyte colony–stimulating factor (G-CSF), commit to neutrophil generation by turning into myeloblasts, which then follow a maturation process that includes the stages of promyelocyte, myelocyte, metamyelocyte, band cell, and finally a mature Ly6G^hi^CXCR4^hi^CXCR2^lo^ neutrophil. In healthy mice, both monocyte populations have short half-lives in circulation, including approximately 19 hours for inflammatory and approximately 2.2 days for patrolling monocytes (132), similar to what was shown for human monocytes, with the exception of a slightly longer lifespan for patrolling monocytes of approximately 4–7 days (133). Human neutrophil lifespan is estimated to be approximately 19 hours (134) to 5.4 days (135), and for mice approximately 12 hours (136). (**B**) Spatial distribution of myeloid subsets in GBM TME. Schematic illustration of the presence of various myeloid subsets in specialized areas of the TME, including perivascular, perinecrotic, and invasive edges of GBM. The neutrophil versus monocyte ratio of 1:7 in GBM is the opposite of their presence in blood (7:1). Neutrophils are predominantly localized in the necrotic core, and monocytes and monocytes that have differentiated into monocyte-derived macrophages are in perivascular and perinecrotic areas, while the majority of microglia are in the invasive edge of tumors.

Myeloid cells — granulocytes (including neutrophils, eosinophils, basophils, and mast cells), monocytes, macrophages, and dendritic cells (DCs) — play essential roles in antimicrobial defense, tissue repair, and various inflammatory conditions, including cancer. In the bone marrow, hematopoietic stem cells continually give rise to common myeloid progenitors, which subsequently give rise to granulocyte-monocyte progenitors, eventually producing monocytes and neutrophils as illustrated in Figure 2A.

Neutrophils are retained in the bone marrow by stromal cell–derived factor 1 (SDF-1, or CXCL12) and its receptor on neutrophils, CXCR4 (19). Decreased expression of CXCR4 in combination with increased expression of CXCR2 triggers neutrophil mobilization from the bone marrow into circulation (20). Neutrophils are the most abundant white blood cell in human blood circulation, but not in mice. Human blood consists of about 50% to 70% neutrophils and 30% to 50% lymphocytes, while mouse blood consists of about 10% to 25% neutrophils and 75% to 90% lymphocytes. Regardless, in both humans and mice, neutrophils are the most abundant myeloid cells in circulation; their ratio to monocytes is approximately 7:1 to 10:1 (21, 22). Interestingly, healthy mouse bone marrow contributes to the removal of neutrophils from circulation (23), and increased CXCR4 expression in a subset of circulating neutrophils was identified as a driving mechanism for their return to bone marrow for elimination (24). Neutrophils, as the first line of defense of the body, are constantly screening for microbial infections in the blood circulation. Neutrophils rapidly trap and eliminate pathogens on contact either through phagocytosis, degranulation (granules with a large variety of cytotoxic material), or release of DNA–microbicidal protein complexes in the form of neutrophil extracellular traps (discussed in detail in ref. 25). Research in recent years showed that neutrophil functions extend beyond microbial defense, including roles in tissue repair (26) and cancer (27).

Three major monocyte populations have been characterized in humans: classical (CD14^++^CD16^−^), intermediate (CD14^+^CD16^+^), and nonclassical (CD14^+^CD16^++^) monocytes (28–30). In mice, two subsets are known: inflammatory monocytes (also referred to as classical), which express high levels of Ly6C and CC chemokine receptor (CCR2^+^) (31), and patrolling/circulating (nonclassical) monocytes, which express low levels of Ly6C and lack expression of CCR2. The existence of a rare intermediate monocyte population was shown in humans and mice, but further investigations are required to determine whether they are a distinct population or an intermediary during nonclassical monocyte formation from a classical precursor (32). Ly6C has no clear ortholog in humans; however, CD14 and CD16 are similarly differentially expressed in mouse monocyte subsets and, despite their lower expression, were deemed appropriate for both species in combination with CD115 as monocyte specific (33). Although a comparison of gene expression patterns in monocyte subsets in humans and mice identified common pathways, unique species-specific expression patterns were also detected (33). Further studies are needed to determine whether species’ unique expression patterns can drive distinct monocyte functions in health and disease contexts. Similarly, earlier studies identified differences in tumor-associated macrophages (TAMs) in humans versus mice, but it is worth emphasizing that many of these differences can be affected by dexamethasone (DEX) given to human GBM patients. DEX inhibits major proinflammatory pathways in TAMs (34), and TAM expression patterns were previously normalized to naive microglia in humans, even though TAMs originate from both microglia and blood-derived monocytes (35). Extravasation of monocytes from bone marrow is regulated by a well-known chemokine family named monocyte chemoattractant proteins (MCPs), which include MCP-1 (CCL2), MCP-2 (CCL8), MCP-3 (CCL7), and MCP4/5 (CCL13 in humans, CCL12 in mice). MCPs, especially MCP-1 and MCP-3, attract monocytes through activation of their cognate receptor CCR2 (31) and have been shown to be essential for monocyte mobilization from the bone marrow into circulation (36, 37). Ly6C^lo^CCR2^–^ patrolling monocytes, often referred to as “macrophages in the blood” owing to their more differentiated stage, act within vasculature, surveying the luminal surface of endothelium for injuries and coordinating its repair by recruiting neutrophils as a partner (38, 39). Ly6C^hi^CCR2^+^ inflammatory monocytes are rapidly recruited to sites of inflammation and sites of tissue remodeling, where they extravasate, give rise to monocyte-derived DCs and MDMs, and have been shown to be the major source of TAMs in GBM (40).

## Monocytes and neutrophil recruitment and homing in GBM

Owing to their abundant presence in GBM, monocytes and brain-resident microglia have been subject to intense research for decades. Initially, they were grouped together as one population based on research suggesting a common origin. Eventually, definitive studies on the origin of tissue-resident macrophages came to light, starting with seminal research on the origin of microglia, which showed they originate from yolk sac progenitors during embryogenesis (41–43) and possess high longevity and self-renewal capability (44). In addition to microglia, the homeostatic brain contains border-associated macrophages in the border regions (45), which originate from bone marrow (45, 46). While in homeostatic conditions microglia proliferate at low rates (44, 47), it has been demonstrated using a mouse model of GBM that approximately 70% of microglia proliferate in the TME (48). Recent studies using lineage tracing (40), live imaging (49), and scRNA-Seq demonstrate that activated microglia in GBM mainly accumulate at tumor margins in both murine and human tumors (50, 51).

In general, myeloid recruitment from blood circulation involves interactions between the myeloid cells and endothelial cells (ECs) of the vessel, and occurs through a series of steps including myeloid capture, rolling, adhesion, and intraluminal crawling, and finally myeloid transendothelial migration. There is compelling evidence that the tumor vasculature can curb trafficking of myeloid cells by influencing each step of their recruitment process. Various aspects of monocyte and neutrophil recruitment have been studied in other cancers, but not much is known in GBM (52). Highly motile CX3CR1^lo^CCR2^hi^ monocytes have been shown to infiltrate murine GBM models (40), where they rapidly transitioned to stationary CX3CR1^hi^CCR2^lo^ and CX3CR1^hi^CCR2^–^ MDMs in perivascular areas adjacent to ECs and pericytes (49). Several reports catalogued macrophages in GBM into two functionally extreme states, often referred to in the literature as polarizations: M1 and M2, which differ in terms of receptor expression, effector function, and cytokine and chemokine production (53–55). Other reports using murine tumors and human GBM patient samples illustrated that TAMs are dynamic, and their heterogeneity and multidimensional biology cannot be represented with an oversimplified M1 and M2 dichotomy (35, 56, 57). Literature on newly diagnosed versus recurrent human GBM myeloid composition produced mixed results. One study using scRNA-Seq showed that MDMs are major infiltrates in pretreatment GBM (50). Another study using scRNA-Seq, together with cellular indexing of transcriptomes and epitopes by sequencing (CITE-Seq) in limited and unmatched primary and recurrent GBM patient samples, showed that primary tumors have a higher abundance of microglia, while recurrent tumors show a higher abundance of MDMs (58). Discrepancies in these studies can be largely attributed to use of marker combinations to distinguish various myeloid subsets and to limited patient samples that do not sufficiently represent the heterogeneity of GBM. A large human glioma scRNA-Seq data set, which could be consolidated from existing data sets, would be a useful resource for the wider GBM and tumor immunology community and would help to address caveats of limited sample sizes.

Although there is no research describing driving mechanisms of neutrophil infiltration into human GBM, limited literature on neutrophil localization in GBM showed that neutrophils colocalize with necrosis temporally and spatially (59) (Figure 2B). Neutrophils are typically mobilized and recruited to sites of inflammation by ELR motif–containing CXC (ELR-CXC) chemokines (ELR motif is the glutamic acid–leucine–arginine sequence), such as murine CXCL1, CXCL2, and CXCL5 and human CXCL8 (also known as IL-8), which binds to the neutrophil receptor CXCR2 (60). For example, during viral encephalitis in response to IL-1α, astrocytes and neurons produce the neutrophil recruitment chemokine *Cxcl1*, which is essential for CXCR2^+^ neutrophil trafficking into inflamed CNS (61, 62). Similarly, it was shown that G-CSF and CXCL1 act synergistically to promote neutrophil mobilization during experimental autoimmune encephalitis, which led to deterioration of the clinical course. In multiple sclerosis (MS) patients, CXCL1, CXCL5, and neutrophil elastase correlated with measures of MS lesion burden and clinical disability (63). These results collectively suggest that during neuroinflammation, neutrophils can infiltrate into the CNS, and their increased presence is associated with CNS disease severity. In contrast to many neutrophil-enriched tumors, GBM is characterized by a low presence of neutrophils. Therefore, neutrophils have been largely ignored within the context of GBM.

Why GBM is highly infiltrated by monocytes and not neutrophils is an intriguing question that remains to be answered. Does it depend on tumor location, driver mutations, or both? Can oncogenic drivers or tumor suppressors dictate what type of myeloid cells are recruited via regulation of expression of chemokines and cytokines responsible for their recruitment? Or does the unique blood-brain barrier or other unaccounted-for factors contribute to this phenomenon? These are essential questions to answer in light of increased awareness of both the genetic and molecular heterogeneity in GBM. In this regard, oncogenic RAS, which is the most common mutation in human cancers (~19% of human cancers harbor a RAS mutation; ref. 64), has been shown to induce IL-8 expression to increase neutrophil recruitment to tumor sites (65). Similarly, in an oncogenic *Kras^G12D^*–driven mouse model of lung cancer (66) and pancreatic ductal adenocarcinoma (67, 68), neutrophils were shown to be a major population of tumor-immune infiltrates. These results clearly illustrate that oncogenic driver mutations can drive neutrophil recruitment and raise questions as to whether the recruitment and function of neutrophils in tumors can be impacted by other driver mutations or common tumor suppressors. While genetic alteration of RAS is rare in adult human GBM (accounting for ~1% of cases; refs. 69, 70), RAS can be activated as a downstream target of amplifications/mutations in EGFR (amplified or mutated in 45% and 26% of GBM patients, respectively), PDGFRA (13%), ERBB2 (8%), and MET (4%), or deletion or mutation of its negative regulator NF1, which is found in about 10% of GBM patients (6). In a study using multiple driver mutations induced in genetically engineered mouse models of GBM, *Nf1*-silenced tumors, although they had an increase in total myeloid cell infiltration, showed lower monocyte presence and had increased numbers of microglia and neutrophils. This is in contrast to *EGFRvIII*- and *PDGFB*-driven tumors, which had greater monocyte infiltration but fewer microglia and neutrophils present (56). Increased myeloid presence was also documented in the human MES GBM molecular subtype, where most NF1-deleted/mutated tumors are clustered together (9), and in the MES cellular state (18). FACS analysis also demonstrated greater microglia abundance in the MES human GBM subtype (57), which is interesting since the MES signature is associated with invasive phenotypes at the tumor margin, which is a microglia-enriched area in both murine and human GBM (40, 50, 51). In a mouse model of EGFR-driven GBM, it was shown that temozolomide treatment at high doses decreases myeloid infiltration from blood and increases microglia presence (71). Another study using *Hras^G12V^*- and PDGFB-driven de novo mouse GBM models showed that *Hras^G12V^*-driven GBMs have an increased neutrophil presence. Similarly, the MES human GBM subtype shows increased expression of neutrophil recruitment chemokines (72).

## The role of neutrophils in GBM progression

Through the use of various experimental methods in many cancer types, some studies have shown that neutrophils promote tumor growth and metastasis via multiple mechanisms, including cytokine production, regulation of angiogenesis (73), and facilitating pro-metastatic niche formation and metastatic progression (74, 75). Paradoxically, others have shown opposite effects of neutrophils (76–78). Subsequent data demonstrated that ratios of pro- and antitumorigenic neutrophils shift during disease progression in a context-dependent manner (79). The phenomenon of functional plasticity and heterogeneity of neutrophils in a cancer context–dependent manner is applicable to other myeloid cells, including tumor-associated macrophages and monocytes (3). Although studies in 1999 to 2000 showed a strong correlation between glioma grade and neutrophil recruitment, with their highest presence in GBM and circulation (80), neutrophil research in GBM has remained in its infancy until very recently. A study with a limited human GBM patient cohort suggested that increased pretreatment neutrophil lymphocyte ratio is associated with poor prognosis in GBM patients (81). Neutrophils promote glioma growth via induction of S100A4 expression in glioma cells, and S100A4 deletion increased the efficacy of anti-VEGFA therapy in tumor-bearing mice (82). Another study suggested that enhanced neutrophil activity was associated with increased levels of IL-12p70 and correlated with worse patient outcome (83). In addition, neutralizing neutrophils by using a monoclonal antibody against Ly6G resulted in increased survival of an *IDH1*–wild-type (WT) glioma model, but not an *IDH1*-mutant (MUT) PDGFB-driven glioma mouse model. Interestingly, *IDH1*-MUT gliomas, which are less aggressive than *IDH1-*WT gliomas, have low tumor neutrophil infiltration and downregulation of chemotaxis-related genes (84). Another study showed that in contrast to *IDH1-*WT, neutrophils in *IDH1-*MUT glioma were not immunosuppressive, owing to increased expression of G-CSF by cancer stem-like cells as a result of epigenetic reprogramming (85). In glioma cell and human peripheral blood neutrophil cocultures, glioma cell–derived IL-6 and IL-8 extended neutrophil survival, suggesting that glioma cell–neutrophil interactions are reciprocal (86). Another study showed that neutrophils isolated from mouse gliomas killed tumor cells in cocultures, while in vivo killing of tumor cells by ferroptosis induced necrosis, which was associated with a MES transition and poor outcome of patients (59). These results were further confirmed by another study showing that the most aggressive GBM subtype, MES GBM, has a higher neutrophil-related gene expression profile (72).

An important question remains: why is a rare population in circulation, such as monocytes, highly recruited into GBM, while the most abundant myeloid cell population in circulation, neutrophils, are rare infiltrates in GBM, with the exception of the aggressive MES GBM subtype (72)? For example, in response to microbial challenge, tissue-resident macrophages produce neutrophil chemoattractants such as CXCL1, CXCL2, and IL-1α, which results in rapid recruitment of neutrophils to the site of infection (87). Now it is clear that the interaction of monocytes and neutrophils in the context of GBM is not following patterns observed in microbial infection; instead, monocyte recruitment appears to occur at the exclusion of neutrophil recruitment. Interestingly, in mouse models it is documented that *Nf1*-silenced tumors exhibit increased numbers of both microglia and neutrophils, and fewer monocytes (56). Similarly, increased microglia presence was shown in MES human GBM (57). This raises the unanswered question of whether brain-resident microglia behave like tissue-resident macrophages during microbial infection and recruit neutrophils. It should be determined experimentally whether these findings are merely coincidental or whether there are microglia-specific neutrophil recruitment mechanisms at play.

## Monocytes, neutrophils, and non-neoplastic cells interact in TME

Although initially defined in cancer, it is now appreciated that pathological conditions that cause inflammation, including infections, can partially block myeloid progenitors and immature myeloid cells from fully maturing into monocytes or neutrophils. These conditions result in expansion of cell populations, which gain distinct phenotypes characterized by the ability to suppress T cell responses (88). In the literature there are two main subtypes of these cells based on their phenotype and morphology — monocytic myeloid-derived suppressor cells (M-MDSCs) and granulocytic or polymorphonuclear MDSCs (PMN-MDSCs) — which exhibit different immunosuppressive properties (89). Most preclinical studies use the markers Gr1 (which captures both Ly6C- and Ly6G-positive monocytes and neutrophils) and CD11B for MDSCs, and Ly6C and Ly6G for further discrimination of M-MDSCs (Ly6C^+^Ly6G^–^) from PMN-MDSCs (Ly6C^+^Ly6G^+^). In humans, M-MDSCs are defined as CD14^+^CD15^–^HLA-DR^lo/–^ and PMN-MDSCs as CD11b^+^CD14^–^CD15^+^CD66^+^ (for more details see ref. 90). These marker combinations are also used to define monocytes and neutrophils in circulation and tumors; therefore, for accuracy and consistency we will refer to the aforementioned cells as monocytes and neutrophils in the tumor context for both humans and mice.

## Neutrophil/monocyte–T cell interactions

The mechanisms by which neutrophils and monocytes achieve T cell immunosuppression are diverse and can result in either a direct or an indirect effect on T cell properties.

### Oxidative stress and nutrient depletion.

**Figure 3:**
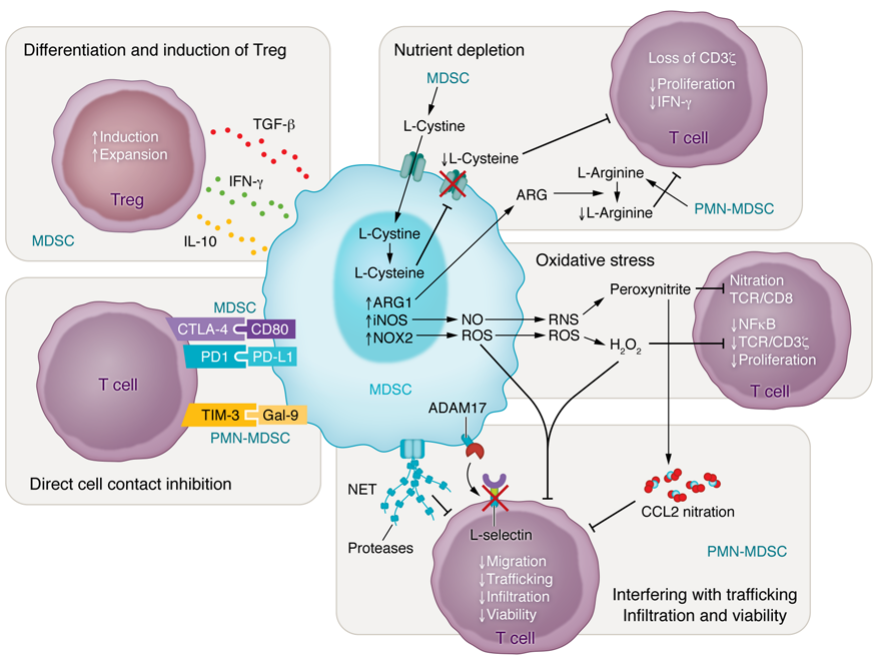
Myeloid-mediated mechanisms of T cell immunosuppression. Myeloid cells have developed several mechanisms to induce T cell suppression, including the induction/expansion of Tregs (top left), direct inhibition via cell contact (bottom left), depletion of nutrients from the tumor microenvironment (top right), induction of an oxidative stress state (middle right), and interfering with trafficking and infiltration and decreasing the viability of T cells (bottom right). The labels MDSC and PMN-MDSC in navy indicate whether studies of the mechanism shown were specific for PMN-MDSCs.

Neutrophils and monocytes secrete high levels of reactive oxygen species (ROS) and reactive nitrogen species, which result in the upregulation and expression of arginase 1 (Arg1) and inducible nitric oxide synthase (iNOS), respectively. Peroxynitrite leads to the nitration of tyrosines in the T cell receptor (TCR)–CD8 complex, which might trigger a conformational change affecting the interaction with peptide-loaded MHCI, rendering the CD8^+^ cytotoxic T cells unresponsive to antigen-specific stimulation (91). ROS production is mainly mediated by NADPH oxidase 2 (NOX2) in neutrophils and has several functional consequences. Hydrogen peroxide (H_2_O_2_) can suppress T cell activation and proliferation by decreasing NF-κB activation (92), downregulation of TCR:CD3ζ chain (93), and inhibition of cytokine production (94). Release of high levels of proapoptotic ROS such as H_2_O_2_ and NO mainly by neutrophils and monocytes can also lead to early induction of T cell death (95). In GBM, neutrophils are the main population expressing and producing arginase (96). Both arginase and iNOS are able to catabolize l-arginine, a dibasic cationic amino acid that plays a central role in the regulation of T cell functions. Depletion of l-arginine also results in downregulation of TCR surface expression by decreased CD3ζ chain biosynthesis. This in turn limits activated T cell proliferation and secretion of IFN-γ (72, 96, 97). T cells also depend on exogenously generated cysteine to support their activation and proliferation. Monocytes and neutrophils express cystine transporters so they can acquire cystine from the TME, but they do not express the transporter to export cysteine. Therefore, these cells sequester cysteine and reduce the cysteine concentration available in the microenvironment, which in turn blocks T cell activation (98) (Figure 3).

### Interfering with T cell trafficking, infiltration, and viability.

Neutrophils can downregulate L-selectin expression on T cells through their cell surface expression of ADAM17 (a disintegrin and metalloproteinase domain 17), an enzyme known to cleave the ectodomain of L-selectin. Downregulation of L-selectin on CD4^+^ and CD8^+^ T cells impairs antitumor activity by interfering with their migration and minimizing the access of T cells to the TME (99). In addition, intratumoral production of peroxynitrite can lead to nitration of the CCL2 chemokine, limiting T cell trafficking within the tumor and resulting in trapping of the cytotoxic T lymphocytes in the stroma surrounding cancer cells (100). In cancer, neutrophil extracellular traps (NETs) from neutrophils can also function as a physical barrier to “protect” tumors from infiltration of immune cells and limit the contact between cancer cells and T cells (101). NETs can not only shield tumors from attack by T cells by physically separating the immune cells from the cancer cells, but other proteins embedded in the NETs can result in an immunosuppressive outcome. Proteases such as neutrophil elastase, myeloperoxidase, and other antimicrobial proteins and factors can be detrimental for T cell function, for example by proteolytic degradation of proinflammatory cytokines (102).

Finally, additional mechanisms that require cell-cell contact have been reported to affect T cell viability. Hypoxic conditions and production of IL-10 in the TME can lead to upregulation of PD-L1 on neutrophils and monocytes (103). Expression of PD-L1 and CD80 (ligand of the immunosuppressive CTLA-4 checkpoint on T cells) was strongly enhanced in neutrophils and monocytes infiltrating GBM compared with splenic counterparts (104). Direct neutrophil/monocyte–T cell contact and interactions between immune checkpoints on T cells and checkpoint ligands on myeloid cells impair antitumor immune responses. These interactions can result in either T cell–induced apoptosis or T cell exhaustion, and a detailed discussion of the mechanisms is provided elsewhere (105, 106). Another contact-dependent mechanism involves interaction between galectin 9 expressed on neutrophils and TIM3 on lymphocytes, binding that triggers cell death in T cells (107).

### Induced differentiation and expansion of Tregs.

Proper effector functions of T cells are counteracted by immunosuppressive lymphocytes such as induced regulatory T cells (iTregs). Myeloid subsets can contribute to the development and induction of naive CD4^+^ T cells to differentiate to iTregs by secretion of cytokines such as IL-10, IFN-γ, and TGF-β in a mechanism independent of NO production (108). Tumor-derived myeloid cells were also shown to selectively expand preexisting pools of iTregs (109). It is worth mentioning that several processing methods for isolation of myeloid cells have been described, including density gradients, magnetic beads, and flow cytometry sorting. These methods can differentially impact the phenotype and functionality of these cells and should be considered and standardized (110, 111).

In addition to interacting with cells of the adaptive immune system, studies have also demonstrated that myeloid subsets interact with other nonimmune, non-neoplastic cells in the TME, especially in perivascular niche areas. Among the nonimmune cells that compose the tumor perivascular niche are ECs, pericytes, and astrocytes (5, 112). Although most monocytes that infiltrate GBM are CCR2 positive, previous subsets present in circulation that are negative for CCR2 and positive for the angiopoietin receptor TIE2 (TIE2-expressing monocytes, or TEMs) have been shown to also infiltrate GBM and exhibit potent proangiogenic activity (113). GBM is a highly angiogenic tumor, partially depending on VEGF to drive angiogenesis and vascular permeability. Upon tumor infiltration, myeloid cells participate in reciprocal interactions with ECs to further promote angiogenesis by multiple mechanisms, including via production of VEGFA (114). GBM is also enriched with reactive astrocytes, most of which are localized either in peritumoral areas with microglia or in perivascular areas with TAMs (115). Reactive astrocytes are less studied than myeloid cells; however, several studies have shown that they promote tumor growth and invasion in primary (115, 116) and metastatic brain tumors (117). These results highlight the multifaceted relationship that myeloid cells have with not only tumor cells, but other non-neoplastic cells in the TME to promote tumor progression. Questions yet to be answered include: Do various myeloid subsets interact with each other? How do they interact with neurons, pericytes, and other non-neoplastic cells in the GBM TME? Novel emerging technologies, including scRNA-Seq in combination with spatially resolved transcriptomics (stRNA-Seq) together with spatially resolved metabolomics, all combined with artificial intelligence, will open new avenues for potential therapeutic exploitations.

## Myeloid-targeted therapies in GBM

Despite intensive efforts over decades, we have learned that it is extremely difficult to therapeutically modulate tumor-associated myeloid cells. This is in large part because we still lack a complete understanding of the heterogeneity that exists among the tumor-associated myeloid compartment in the context of inter- and intratumor heterogeneity of GBM. CSF1R inhibition was shown to be effective in targeting TAMs and prolonging survival in a PDGFB-driven adult glioma mouse model (54); however, in contrast to elimination of about 95% of microglia in naive mice (118), tumor-infiltrating myeloid cell numbers were not affected, but rather their expression signature was changed. These results suggest that TAM survival is independent of CSF1R. Unfortunately, CSF1R inhibition failed to demonstrate effectiveness in a clinical trial with unselected human adult recurrent GBM patients (119). In contrast to a promising preclinical study of CSF1R with radiation therapy (RT) (120), a phase Ib/II clinical trial evaluating the efficacy of the CSF1R inhibitor PLX3397 in combination with RT and temozolomide (TMZ) for newly diagnosed GBM patients compared with robust historical controls found no improvement in median progression-free survival or overall survival (ClinicalTrials.gov NCT01790503) (121). The discrepancies between preclinical and clinical results can be partially attributed to heterogeneity and plasticity in the myeloid compartment of human GBM, which are not fully captured by use of a single mouse model. To better understand tumor heterogeneity and determine whether any given target is pan-myeloid-effective or whether its efficacy is limited to certain genotypes, it would be essential to include other murine models resembling MES and CL human GBM subtypes as well. In a comprehensive study, administration of the CSF1R inhibitor JNJ-40346527 in multiple preclinical mouse models of various solid tumors reduced macrophage numbers but failed to stop tumor progression owing to compensatory recruitment of immunosuppressive neutrophils that neutralize the antitumor effects of CSF1R inhibitor (122). This cellular resistance mechanism was not shown in the case of CSF1R inhibitor use in a GBM mouse model (54), likely because of its inability to decrease TAM numbers, which might be necessary for neutrophil compensation to take place. It will be interesting to see whether this mechanism of compensatory neutrophil infiltration takes place only in response to CSF1R inhibitors or is a general cellular resistance mechanism that occurs when the infiltration of one myeloid subset is decreased, leading to compensatory recruitment of others. The latter case could have important clinical implications for therapies targeting a single myeloid subset.

In addition to CSF1R, the established pro-tumorigenic and immunosuppressive functions of CCR2^+^ monocytes have prompted evaluation of CCR2/CCL2-targeted therapies in GBM. Neither genetic deficiency of CCR2 nor a small-molecule inhibitor of CCR2 (CCX872) showed any efficacy in extending the survival time of tumor-bearing mice compared with WT or vehicle-treated mice, despite significant reductions of CCR2^+^ monocyte and CD45^+^CD11B^+^ myeloid infiltration into tumors (123). In contrast, another study using GL261 gliomas in *Ccr2^–/–^* mice showed that although monocyte recruitment was significantly impaired, the total number of macrophages was not affected and correlated with compensatory microglia proliferation (58). Although ineffective as a monotherapy, when combined with anti–PD-1, anti-CCR2 provided potent antitumor effects in two syngeneic GBM models, suggesting that targeting of CCR2 and PD-1 is a therapeutic combination worth exploring in human GBM patients. The question remains of whether Ly6C^+^ monocytes that still infiltrate into GBM in CCR2-deficient tumor-bearing mice express CCR2 and whether a subset exists that does not express CCR2, thereby negating any positive survival benefit in tumor-bearing mice. Other compensatory mechanisms might be responsible for persistent recruitment of monocytes into GBM. Genetic deficiency of CCL2 in mouse models of GBM resulted in extended survival of tumor-bearing mice, but did not result in a significant reduction of macrophage presence in tumors (40). Another study using GL261 and xenograft GBM models showed that systemic administration of anti-CCL2 antibodies resulted in significant reduction of both CD45^+^CD11b^+^ and CD11^+^Gr1^+^ myeloid cells and modestly increased the survival time of tumor-bearing mice, while remarkable anticancer efficacy was achieved when anti-CCL2 antibodies were combined with TMZ (124). It must be experimentally investigated whether these differences can be attributed to compensatory signaling mechanisms taking place with other chemoattractant-family chemokines such as CCL7, CCL8, and CCL12, which can all signal through CCR2 (125, 126), or whether these differences are driven by differences in the genetic landscape of the experimental mouse models used.

In addition to promoting tumor progression, myeloid cells have also been implicated in GBM resistance to various therapies, including anti-VEGFA therapy (114, 127). Using a GBM xenograft model, it has been shown that RT increases myeloid infiltration, and that the increased myeloid cell presence is responsible for tumor recurrence (128), similarly to what was reported recently in a PDGFB-driven mouse model of GBM (120). It has also been shown that chemotherapy can induce IL-1β release by myeloid cells, which in turn decreases antitumor efficacy of chemotherapy and promotes tumor growth (129). Overall, these results highlight the model- and tumor-dependent heterogeneity of monocyte recruitment mechanisms and the challenges in completely abolishing tumor infiltration of myeloid cells. It is also clear that reducing myeloid cell infiltration is sufficient to increase the efficacy of immunotherapy and chemotherapy in GBM. Although these studies provide a rationale for combinatory treatment, it is also apparent that further studies are necessary to understand resistance mechanisms emerging from myeloid-targeted therapies. In patients treated with chimeric antigen receptor (CAR) T cells, the frequency of myeloid subsets inversely correlated with the levels of CAR T cells, with higher numbers in patients who did not respond to therapy (130). Optimizing CAR T cells to resist this immunosuppression or targeting the myeloid compartment can boost the efficacy of adoptive T cell therapy.

Until recently, owing to their low numbers and unknown functions, therapies targeting neutrophils for GBM were still in their nascency. Limited studies with neutrophil targeting in GBM have been achieved using anti-Ly6G neutrophil-depleting antibodies. Using anti-Ly6G antibodies at early and late stages of tumor development in genetic mouse models, one study showed that neutrophils exhibit antitumor roles at early stages and pro-tumorigenic roles at later stages (72). Another study using human GBM xenografts showed antitumor efficacy of anti-Ly6G antibody treatment, even though rebound effects are known to exist, with neutrophils eventually becoming resistant (59, 131). More research is needed to better understand the role of neutrophils in GBM and their interaction with other myeloid and adaptive immune cells in the GBM TME.

Table 1 summarizes preclinical evaluation of monocyte-, TAM-, and neutrophil-targeted strategies alone and in combination with other therapies.

## Perspectives

Remarkable progress has been made in characterizing the genomic landscape of GBM, and we have come to appreciate that it is a highly heterogeneous tumor with an equally complex and heterogeneous TME. Although decades of research has been dedicated to understanding the role of major immune infiltrates in GBM, such as monocytes and monocyte-derived macrophages, targeting these cells has proven to be very challenging due to multiple complex mechanisms. First, genetically stable myeloid cells evolve with tumor progression, and pathways important at the initial stages of tumor development might not be necessary at later stages of tumor progression. Second, tumor cells with heterogeneous cellular states and driver mutations within the same tumor are plastic and are influenced by infiltrating myeloid cells. Specific pathways important in one GBM TME might not be necessary in other GBM TMEs with differing genetic constituencies. Therefore, for targeting a specific pathway in a subset of myeloid cells, it is essential to determine whether treatment efficacy is dependent on tumor genotype or specific cellular states of the tumor. While it is essential to understand the role of myeloid subsets in tumor initiation or early stages of tumor development in mice, for therapies to move to clinical trials in human GBM patients it is also essential to evaluate their efficacy in mice with apparent medium to large tumors.

Understanding the interconnected mechanisms between monocytes and neutrophils becomes essential not only for GBM but also for other cancers. Moving forward, considerable efforts must be made to determine the biological relevance of these heterogeneous cellular interactions using proof-of-principle validation approaches and multiple in vivo GBM models. These efforts will enable the field to develop more efficacious myeloid-targeted therapies or use myeloid cell targeting to increase the efficacy of standard-of-care and novel emerging immunotherapies.

## Figures and Tables

**Table 1:**
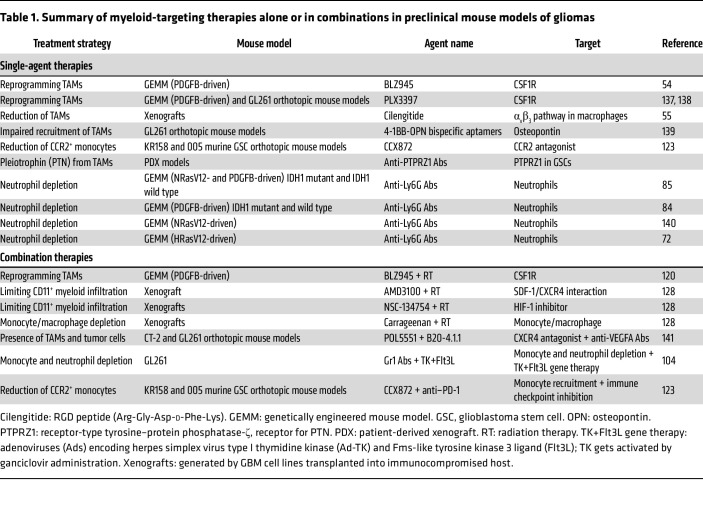
Summary of myeloid-targeting therapies alone or in combinations in preclinical mouse models of gliomas

## Version 1 01/03/2023

Electronic publication
